# Support System as a Foundation for Self-Care Management Among Heart Failure Patients in Saudi Arabia

**DOI:** 10.7759/cureus.116737

**Published:** 2026-09-22

**Authors:** Noor Ezah Binti Mahno, Lee Siew Pien, Hanida Hani Binti Mohd Mokhtar, Hazem Amer, Rawnaq B Al Amri

**Affiliations:** 1 Nursing, International Islamic University Malaysia, Kuantan, MYS; 2 Nursing, King Fahd Military Medical Complex, Dhahran, SAU; 3 Interventional Cardiology, King Fahd Military Medical Complex, Dhahran, SAU

**Keywords:** caregiver, healthcare provider, heart failure, self-care management, support system

## Abstract

Background and objective

Various support system interventions have demonstrated efficacy in enhancing self-care management among patients with heart failure (HF) through a comprehensive understanding derived from diverse perspectives involving patients, caregivers, and healthcare providers (HCPs) in self-care practices. The objective of this study was to explore the self-care management needs of patients with HF and how caregivers and HCPs support these needs.

Methods

A qualitative approach was used to examine patients’ experiences with HF. Purposive sampling was used to recruit 24 patients from a Saudi tertiary center between May 2024 and May 2025. Data collection involved semi-structured, face-to-face interviews about their understanding, practices, and challenges. The interviews were recorded, transcribed, and analyzed thematically.

Results

The findings highlight the importance of support systems as a foundation for self-care management among patients with HF. Participants with HF reported that their spouses were their primary caregivers. Most were satisfied with the care and communication provided by HCPs. Many did not exercise because of their condition. Most reported that HF-related costs were covered by the government, with some receiving financial aid. Others relied on family and relatives for support.

Conclusions

Support systems serve as a vital foundation for self-care management among patients with HF. Family caregivers, HCPs, and institutional resources all play crucial roles in ensuring comprehensive self-care management for patients with HF. Social media, newspapers, education initiatives, and free health screenings serve a significant role in raising public awareness and understanding of risk factors that predict HF.

## Introduction

Heart failure (HF) is a leading cause of disease worldwide and represents one of the most common diagnoses associated with high rates of hospital readmission and mortality. Recent studies by Liu et al. have demonstrated that HF self-care management not only improves survival rates but also enhances quality of life across physiological, psychological, and social domains [1]. Therefore, instead of continuously struggling with unpredictable exacerbations of HF symptoms, patients with HF need to find value in living and greater motivation to survive. Alhamory et al. reported that healthcare challenges perceived by patients include the burden of treatment faced by patients with HF, which might trigger psychological distress. These challenges include seeking a human touch, long waiting times, insufficient health information and healthcare resources, and the involvement of mental health specialists to help understand their distress [2].

Managing self-care in patients with HF requires recognizing early signs and symptoms as they appear. It is fundamental to chronic disease management, such as HF, and can greatly enhance treatment success while reducing the impact of HF [3]. Proper self-care is vital for effectively managing HF. Patients who engage in positive self-care behaviors tend to better regulate their condition, reduce hospital readmissions, lower healthcare costs, and achieve a higher quality of life. Massouh et al. demonstrated that patients with HF benefit from self-care management, with improved outcomes linked to increased knowledge, confidence, and caregiver support [4]. Evidence shows that self-care for HF includes activities aimed at maintaining health and making decisions to control symptoms. Worsening symptoms are a primary reason for readmission [5]. Adequate self-care support from caregivers, healthcare teams, social services, and systemic resources is crucial to enhancing self-care management in these patients.

Several studies indicate that patients with HF need adequate social support, and active participation in social networks can reduce loneliness and stress associated with living with HF. Another study by Liu et al. stated that patients with HF received social support from both family and healthcare providers (HCPs), which alleviated some care needs during the chronic phase and improved health outcomes [1]. A study demonstrated that providing consistent social support through regular clinic visits, home visits, and telephone follow-ups for appointment reminders may enhance patients’ perceived health status [6]. HCPs should provide clear discharge instructions, whether verbal or written, to guide patients with HF in self-care after hospital discharge. These instructions are essential for managing tasks at home, such as medication adherence, healthy eating, lifestyle choices, social support, and routine follow-ups. Failing to comply with discharge instructions can result in poor health outcomes, reduced quality of life, and frequent hospital readmissions.

Consistent health education during follow-up appointments is crucial to prevent patients from developing an inadequate understanding of at-home self-care practices. Research has shown that tailored education and interventions for patients with HF can enhance treatment adherence and boost self-care awareness, resulting in improved habits [7]. Recording unpredictable symptoms in a diary during the chronic phase, after adjusting diuretic doses, provides valuable insights and aids in monitoring symptoms for future treatment adjustments. These methods, as claimed by Sousa et al., including face-to-face and telephone follow-ups, encourage self-care by prompting patients to document symptom changes in diaries [6]. The Heart Failure Program (HFP) offers patient education on Awareness Day, delivered by a team of physicians, dietitians, cardiac rehabilitation nurses, a smoking cessation clinic, and clinical pharmacists who support medication management. Nurses educate patients on early warning signs such as shortness of breath, ankle swelling, rapid weight gain, and chest pain.

A recent study described a comprehensive HF management program that combines educational resources with positive attitude reinforcement and benefits patients [8]. These practices follow the 2021 European Society of Cardiology guidelines, which recommend follow-ups every six months. Evidence-based HFPs have demonstrated benefits such as improved quality of life and slower symptom progression. AlAyoubi et al. found that a multidisciplinary program in Saudi Arabia reduced readmission rates among NYHA class II patients in outpatient clinics, thereby lowering the overall annual cost of HF [9]. Consequently, Middle Eastern countries should adopt more HFPs to reduce the prevalence of the disease. A study found that patients prefer information on self-management of HF, which helps improve their health and reduce hospital readmissions [2]. Recent studies show that several interventions have been implemented to improve HF outcomes, with support systems as a foundation for self-care management, including distance health education [6,7], HF multidisciplinary programs [9], and educational support [10]. Limited studies have been conducted in Middle Eastern countries on patients with HF regarding their perceptions of the need for and support for self-care management, and there is no standardized management of patients presenting with acute HF. These significant gaps highlight the need for a qualitative study that focuses on patients’ perceptions of HF self-care management and support systems.

Objectives

The research objective of this study is to explore the self-care management needs of patients with HF and how caregivers and HCPs support these needs.

## Materials and methods

This study employed a generic qualitative research design. Caelli et al. stated that a generic qualitative research design is a study that seeks to discover and understand a phenomenon, a process, or the perspectives and worldviews of the people involved [11]. This type of research design is simple and interpretive and works well as a clear way for researchers to understand how patients view managing their HF on their own. During interviews, we explored their perceptions, the support they received, and what they need from caregivers and HCPs to help them feel more confident and supported.

Semi-structured, in-depth interviews were conducted with a group of patients with HF from diverse backgrounds at a tertiary hospital in Saudi Arabia. As Bernard’s study suggested, the researcher identified what needed to be known and set out to find people who could provide the information through their knowledge or experience [12]. The researcher identified potential participants by screening data from the health information system and by visiting the selected units to screen potential participants who met the inclusion criteria, following full ethical approval from the ethics committee and the heads of each department. Participants who fulfilled the inclusion criteria were approached, and information about the study was explained to them. Eligible participants were interviewed in person during the visit, and the interview lasted at least 60 minutes. The English versions of both instruments, the information sheet, informed consent form, and interview guide were translated into Arabic using a back-translation method by two independent translators. The topics discussed included the need for interpersonal relationships and support from caregivers and HCPs.

Before conducting the semi-structured, in-depth interviews, an interview guide based on the Adams study was prepared to streamline the process. It consists of specific and open-ended questions designed to encourage participants to share their perspectives on the predetermined topics [13]. Typically, the questions are complemented by follow-up “why” or “how” inquiries to explore their responses in greater depth. Adams’ study noted that a semi-structured, in-depth interview typically involves a single conversation with a participant, lasting about one hour [13]. It directs the conversation between the researcher and participants, with additional follow-up questions, and provides a supporting list of questions to develop better responses from participants. Probes and comments are made to obtain more data. In this study, the interview guide was developed based on literature reviews [14-16] and comprised several specific questions related to perceptions and views of HF, its treatment, and self-care management. The researcher conducted pilot tests with a small group of participants who met the study’s inclusion criteria, received feedback, and revised and edited the interview guide draft before further data collection.

The inclusion criteria for the participant group were age over 18 years, a diagnosis of HF with or without comorbidities, admission to the Coronary Care Unit and Cardiac Ward, Saudi nationality, and fluency in Arabic or English. Patients were excluded if they were critically ill, including those on ventilators and those with hemodynamic instability. A total of 27 participants were interviewed, but only 24 were selected using purposive sampling between May 2024 and May 2025. The researcher assigned each participant a unique number in the records, ranging from Patient 1 to 27. The interviews were conducted at the participants’ own pace and time to ensure that participants felt comfortable during the interviews. Due to unsatisfactory progress, Patient 22 was excluded from the study. Patients 23 and 26 had already been added to the record, but they suddenly discontinued the interview due to unavoidable circumstances. The informed consent form stated that participants had the right to refuse and withdraw from the study at any time. Consequently, only 24 participants completed the interviews, while three patients were excluded.

The data were analyzed using thematic analysis; Creswell stated that it is a systematic process for coding and categorizing data into themes that represent the phenomenon of interest [17]. Ethical approval was obtained from both the university and the respective hospital prior to data collection: the Kulliyyah of Nursing Postgraduate Research Ethics Committee (KNPGRC) and the IIUM Research Ethics Committee (IREC) in Malaysia. Subsequently, approvals were obtained from the Ethics Research Committee at a military hospital in Saudi Arabia and the Institutional Review Board of the Medical Services General Directorate of the Armed Forces Hospitals Eastern Region, Saudi Arabia. Participants were briefed on the recruitment and research process. The information sheet and informed consent form were provided and obtained before the interview sessions. Participants’ confidentiality was maintained by not revealing names or identities during data collection, analysis, and reporting. Interview data were audio-recorded with participants’ permission. All interviews were transcribed verbatim by the researcher. The data were also saved in Word format and assigned pseudonyms to participants on a computer to facilitate analysis. The researcher performed open manual coding inductively and produced preliminary codes to analyze the qualitative data. Four researchers coded the interviews independently and came together to develop a preliminary coding tree and compare all codes, with regular feedback from the research team. The researcher discontinued data collection once data saturation was reached and no new themes or insights emerged from the participants.

Lincoln and Guba stated that trustworthiness in qualitative research depends on credibility, transferability, confirmability, and dependability [18]. The data analysis process included identifying themes, highlighting them, generating initial codes, and labeling these with provisional themes. After individual analysis, peer debriefing with the supervisor ensured that all themes were cross-checked to reach a final consensus. Participants were selected through purposive sampling to explore their perceptions and enhance the transferability of the findings. Initial codes arose from significant topics identified during data collection and informed the review and definition of themes, as well as the rationale for combining codes. The researcher verified transcripts, maintained an audit trail, selected appropriate methodologies, analyzed the data, interpreted the findings, and presented the conclusions. A reflexive journal documented the process, thereby enhancing trustworthiness. The researcher interpreted the findings and verified them through member checking and triangulation. The researcher organized the data in a table to visualize the relationships and facilitate interpretation while maintaining reflexivity about their own biases. The researcher also established a qualitative reporting framework, such as the COREQ checklist by Tong et al., which was followed [19].

## Results

Twenty-four participants agreed to be interviewed during data collection. Table 1 presents background information on the demographic characteristics of patients with heart failure (n = 24). The data collection form for the participants is shown in Appendix A. All participants in this study were Saudi. More than half of the participants were between 51 and 80 years old, with a lower proportion of females (12.5%). The majority were married (91.7%), most patients with heart failure had comorbidities (79.2%), and the majority lived with their families (83.3%). In contrast, a smaller proportion lived alone (8.3%). Most participants were retirees (54.2%), and half (50%) had a university education. Appendix B outlines the semi-structured interview guide. Three main themes emerged: psychological and caregiver support; effective communication and comprehensive health education from HCPs; and accessible social and systemic support for ongoing care.

**Table 1:** Demographic characteristics of the HF patients (n = 24). HF, heart failure

Demographic characteristic		Frequency (n)	Percentage (%)
Age	30-50	9	37.5
	51-80	15	62.5
Marital status	Married	22	91.7
	Divorced	2	8.3
Diagnosis	HF	5	20.8
	HF and comorbidities	19	79.2
Race	Saudi	24	100
Gender	Male	21	87.5
	Female	3	12.5
Level of education	Secondary school	12	50
	University	12	50
Employment	Employed	5	20.8
	Unemployed	6	25
	Retiree	13	54.2
Living arrangement	Alone	2	8.3
	Spouse	1	4.17
	Family	20	83.3
	Parent	1	4.17

Theme 1: Psychological and caregiver support in the daily care routine

The support system in this theme refers to the people surrounding the individual who provide emotional and psychological support, especially caregivers. A caregiver provides support to those with short- or long-term illnesses, injuries, or disabilities, helping meet their needs and manage daily activities. They adapt to new living situations while handling emotional distress, work, body changes, financial issues, and family problems. Caregivers play a crucial role in direct care and daily activities, which can impact their own self-care.

Initially, participants in this study were asked about the person who would be taking care of them after discharge home, to reflect on the most recent time they experienced symptoms, and to describe the next steps the caregiver took to prevent worsening of HF symptoms.

One participant said she stays with her children. Although married with children, her son cares for her at home while his wife handles food and medication routines. Every weekend, another family visits her. She feels better emotionally and psychologically, and her health has improved. Patient 27 mentioned that:

*My kids, they are taking good care of me. Every weekend, they (my kids and my grandson) will come and visit me and spend good quality time with me* - Patient 27, 66-year-old female 

Similarly, Patient 20 mentioned that he also has housemaids for assistance, and they have been trained in caregiving roles for routine care:

*My kids will arrange everything for me* - Patient 20, 69-year-old male

Additionally, Patient 12, a taxi driver, admitted that he would not go to work if he felt unwell. His family provides him with financial support:

*I always feel tired and have shortness of breath. Sometimes I don’t go to work every day. If I feel better, I will go to work, and if I don’t, I will ask my family for help* - Patient 12, 61-year-old male

Some participants mentioned that their spouses would be their caregivers, assisting them with activities of daily living, including preparing food, attending to personal hygiene needs, managing medication regimens, and attending follow-up appointments. Patient 9 affirmed that:

*My husband and my kids are my true strength. Alhamdulillah, my husband is aware of my current health status; he is always there to help me. My kids are all grown up* - Patient 9, 43-year-old female

Patient 10 expressed that he has had an autoimmune disease, systemic lupus erythematosus, for several years, and his condition worsened after he was diagnosed with HF. However, his spouse always gives him full support. He said:

*It will be no problem for now; they are with me, taking care of me. My wife, who is a skilled cook, prepared the dishes according to my health needs. My wife is helping me with arranging the medications and reminding me of the follow-up. I can’t walk properly: my right leg still has weakness, and I have to walk using a walker cane* - Patient 10, 50-year-old male

As mentioned above, most participants in this study who received caregiver support reported that it alleviated their burden in terms of emotional, psychological, and financial support, as well as practical needs in daily life, including meal preparation, medication management, transportation, accompaniment for upcoming appointments, and daily caregiving routines.

Theme 2: Effective communication and comprehensive health education

This study highlights that participants’ primary needs are effective communication with HCPs and thorough health education. Consistency and good communication between patients and HCPs are essential throughout the journey after being diagnosed with HF. In this study, most participants expressed their feelings and appreciation for healthcare professionals with enthusiasm, reporting good support in different aspects:

*Good. I understand very well what the doctor had explained to me* - Patient 9, 43-year-old female

*All of them, doctors and nurses, especially Dr. G, are helping me* - Patient 14, 59-year-old male

Some participants reported misunderstandings about the treatment provided by HCPs. Patient 20 stated:

*I do not understand it in English, but in Arabic, of course, even for the doctors also* *- *Patient 20, 69-year-old male

HCPs should use simple language to explain procedures to patients and families who are not medically trained. Most participants emphasized that clear, straightforward communication during consultations is crucial to avoid misunderstandings about medication and self-care responsibilities. Patient 15 mentioned experiencing sudden weight loss and loose stools, along with episodes of forgetfulness over two months:

*They told me about my current condition. My heart valve is not working well; I need to undergo multiple surgeries for my brain, abdomen, and heart valve* - Patient 15, 46-year-old male 

Another form of multidisciplinary support provided by HCPs includes health education and specialized training. According to Patient 19, he had a septal defect since birth. The doctors had explained the implantable cardioverter-defibrillator (ICD) procedure, its complications, and the care required after surgery:

* They said I have to put a battery in my heart* - Patient 19, 31-year-old male

Another participant reported that he understood how to use the incentive spirometry device for his lung exercises and deep breathing, as demonstrated by the respiratory therapist and doctors after his valve surgery in 2019:

*Deep breathing exercise and spirometry exercise after valve surgery *- Patient 15, 46-year-old male

According to the medical record and his wife’s account, Patient 17 used a continuous positive airway pressure (CPAP) machine at night at home due to chronic obstructive pulmonary disease. The respiratory therapist and doctors provided simple instructions before he used the machine.

*I was blind for a long time; I am not so sure when. My wife knows me well and helps me use the breathing assistance machine before sleep* - Patient 17, 48-year-old male

Patient 27 accidentally took a double dose of warfarin over several days due to forgetfulness and miscommunication among her children, leading to an urgent hospital visit. The doctor recommended warfarin education and eating fewer green foods, such as salads, which can affect how warfarin functions in the body:

*The doctor told me especially not to take too much green food* - Patient 27, 66-year-old female

Another participant described the importance of Clexane injections for their condition, as advised by the doctor, and the pharmacist provided health education on administering subcutaneous Clexane at home.

*I have used Clexane injections for one year already - *Patient 14, 59-year-old male

Diabetes educators are HCPs specializing in diabetes care. Before discharge, doctors refer patients with diabetes-related issues to them. They offer personalized support for diabetes self-care, knowledge, and skills. Patient 12 pointed out that:

*Food. I ate anything I wanted. I checked my sugar first and took the insulin as instructed. I have only glucose machine monitoring - *Patient 12, 61-year-old male

Theme 3: Accessible social and systemic support for ongoing care

Social and systemic support for patients with HF involves assistance from family, friends, community groups, and professionals, which improves well-being and helps maintain self-care at home. Patient 5 stated that he received full support from his manager after diagnosis.

*My friends are always there helping me when I need them the most. My work manager is also a very supportive person; he will allow me to take a few months of vacation if I am not feeling well* - Patient 5, 52-year-old male

Most participants reported depression and sleep disturbances, so the doctor referred them to psychiatric care and prescribed antipsychotic medications. According to the medical record, a doctor from another hospital referred Patient 13 for psychiatric consultation after two suicide attempts.

*I am sad. Yes, I am tired of everything… I am the one who is taking care of myself. Nobody does (sad). I am living with my brother and father. My family is always angry with me because I did not take the medications - *Patient 13, 47-year-old male

Raising awareness involves community or institutional resources focused on ongoing care. Some hospitals hold annual World Heart Day events to educate the public, promote preventive actions such as quitting smoking, and encourage healthy habits. These campaigns offer free screenings, consultations, vital sign checks, and health services. Many hospitals also use electronic systems for scheduling, reviewing test results, tracking sick leave, and managing records, making it easier for patients and families to access health information. However, Patient 15 reported that virtual appointments did not provide any benefits for him during the COVID-19 outbreak in 2019:

*I did not skip any appointments, but since the COVID-19 pandemic hit us in 2019, we have had only virtual appointments, which have been so difficult for me during those days. They always delay and postpone my appointments, which makes it hard for me at that time. Sometimes, the supply of medications is also affected; I do not have enough supplies - *Patient 15, 46-year- old male

To achieve Vision 2030, most hospitals offer virtual doctor consultations for medication refills, laboratory test results, and radiology reports, helping patients with transportation issues. Nurses also visit patients at home for follow-ups, with one nurse conducting regular site checkups for certain conditions. For the next appointment, most individuals receive a phone call or text message notification, and the government covers their treatment costs. According to Patient 25, he pointed out that:

*I received an instant SMS for my next appointment. Actually, it is easy for me; I have the privilege to get medical treatment because it’s free for me *- Patient 25, 50-year-old male

Most patients with HF receive full support only from their families. Patient 12 stated that he always feels tired because of his illness:

*I always feel tired and have shortness of breath. Sometimes I do not go to work every day. I don’t really exercise because I have some limitations with my leg, pain, and swelling. If I feel better, I will go to work, and if I do not, I will ask my family for help financially - *Patient 12, 61-year-old male

Most participants prefer to seek financial help from family and relatives due to limited resources. Facing the challenges of the disease, they adapt their routines, manage their relationships, and strive for a work-life balance. Some also receive government support, including a subsidy program for individuals with disabilities. The ministry supports all categories to promote stability and equality.

## Discussion

This study identified a support system comprising caregiver, HCP, social, and systemic support. This encompasses strong psychological support within the family, adequate treatment from HCPs, insufficient discharge education, significant financial aid from the government, and a deficient systemic support system. The systemic gaps perceived by the participants in this study include limited awareness of healthy lifestyles, the high cost of fitness centers, a less organized HFP, and an ineffective online platform during COVID-19.

This study highlights that caregiver support is essential for successful self-care, encouraging positive thinking and improving health outcomes across emotional, psychological, and practical areas for patients with HF. Participants with HF reported that their spouses often serve as their primary caregivers. These caregivers are crucial in daily management tasks such as preparing meals, managing medications, providing transportation, attending appointments, and supporting daily routines. This aligns with a study by Kugler et al., which found that effective self-care management education for patients with chronic HF depends on strict adherence, a supportive family, caregivers, and HCPs to help them understand their lifelong responsibilities [20]. Overall, caregivers play a vital role in reducing the burden on HF patients by ensuring proper management of their condition.

Support from HCPs was vital in this study. Most participants were satisfied with the care and communication, especially when it was face-to-face. This contrasts with research from the Middle East by Skouri et al., which found that half of patients with chronic HF experienced psychological issues and received less HCP support [21]. HF patients often experience psychological challenges due to limited HCP attention. The shortage of HCPs, including doctors, pharmacists, and nurses, leads to insufficient support. Tash and Al-Bawardy highlighted the global shortage of medical staff and called for better education standards and targeted scholarships, especially for nurse practitioners registered with the Saudi Commission for Health Specialties [22]. A lack of professionals and poor system coordination hinder HF patients’ self-care and management. Studies by Tash and Al-Bawardy and Bezerra et al. have shown that fragmented systems, referral issues, and primary care shortages reduce the quality of care, lead to repeated tests, and limit prevention, suggesting that clinicians should focus more on patient-reported measures and mental health to better identify the psychological impact of HF [22,23]. In this study, some participants reported strong support from HCPs, shared hope, increased adherence, and optimism for a quick recovery, especially after committing to long-term treatment and following medication regimens. This aligns with another study by Nordfonn et al., which found that HF nurses motivate patients to follow self-care practices, respond to treatment, and enhance safety and self-efficacy [14]. Nurses also play a crucial role in motivating HF patients to follow self-care practices and achieve treatment goals.

In this study, HF participants reported a lack of discharge instructions and education regarding specific treatments such as warfarin tablets, Clexane injections, ICDs, nebulizers, and CPAP machines. They also felt that their education on diabetic self-care management was incomplete. Many patients with HF are typically given a wide array of self-care tasks by HCPs. This finding aligns with a study by Skouri et al. that patient counseling with HCPs helps improve psychological well-being and quality of life and reduces HF-related hospitalizations [21]. Discharge counseling emerges as an area needing enhancement in HF self-care management within this study. This is supported by Bahrodi et al. and Alharrasi et al., who demonstrated that a comprehensive approach is necessary to address self-care neglect and challenges faced by patients with long-term HF, recommending interventions such as left ventricular assist devices, optimized medication therapies, and improved non-drug strategies such as early symptom detection [24,25].

In this study, HCPs reportedly advised patients to reduce consumption of green leafy vegetables to protect warfarin functionality. Similarly, Mgala et al. found that warfarin users were advised to restrict vitamin K-rich foods, especially green vegetables [26]. Since warfarin works as a blood-thinning medicine that helps prevent blood clots by blocking vitamin K, consuming fluctuating amounts of spinach, broccoli, cabbage, or lettuce can affect international normalized ratio (INR) levels and reduce drug efficacy [27]. Rather than practicing strict food avoidance, patients on warfarin require a consistent daily intake of vitamin K to promote long-term INR stability.

Most participants suffered from depression and sleep disturbances. A similar finding indicates that depression affects approximately 20-50% of patients with HF, with symptoms such as fatigue and sleep disturbances occurring at higher rates with increased severity of HF [28,29]. Living with HF is a heavy physical and emotional burden, which most participants experience as depression. Unfortunately, as HF progresses and physical health declines, the risk of depression increases. At the same time, depression makes it harder to manage HF symptoms, leading to a faster decline in overall health. Recognizing these signs early is crucial because treating depression can significantly improve a patient’s physical and emotional health and quality of life. Another related finding showed that Al Shamiri et al. demonstrated that more than half of the Saudi Arabian HF cohort experienced depression and anxiety [30]. These findings suggest that a strong correlation exists between HF and an elevated risk of developing depression.

Most participants said the government covers HF expenses, which are accessible to all. Healthcare services in government hospitals are free for Saudi citizens and expatriates. Another study by Tash and Al-Bawardy reported that the Ministry of Health (MOH) partners with 26 diabetes or endocrinology centers, 600 smoking cessation clinics, and 24 cardiology centers [22]. These efforts support Saudi Vision 2030 by improving access, care quality, and efficiency, reducing wait times, and promoting disease prevention, aligning with goals to enhance treatment adherence and patient satisfaction for HF self-care. Most participants seek financial help mainly from family and close relatives due to limited personal resources. Faced with disease-related challenges, they often adapt to a “new normal,” which includes changing routines, managing affected relationships, and trying to maintain their lives. Several participants reported receiving financial aid from the government. The Ministry of Human Resources and Social Development offers a financial subsidy program in Saudi Arabia for individuals with disabilities, subject to specific terms and conditions.

Most HF participants reported not exercising due to their medical condition. These findings align with research by Tash and Al-Bawardy, which demonstrated that multiple factors, including gender segregation in fitness facilities, higher expenses, severe weather conditions, and a social media trend promoting high-fat, sugary diets, contribute to the growing sedentary behavior among Saudis, thereby negatively impacting their health and well-being [22]. Patients with HF are concerned that engaging in strenuous exercise could potentially worsen their current exacerbation of symptoms. Therefore, it is not wrong to exercise regularly. However, HF patients need to consult with HCPs to develop a safe and effective exercise plan, as HCPs can assess individual needs and limitations and provide personalized recommendations. The MOH may also utilize social media platforms to promote a healthy lifestyle. HCPs and medical societies are responsible for educating the public regarding the advantages of physical activity and advocating healthy lifestyles among Saudis, as they play an essential role by conducting routine assessments and providing counseling on physical activity and lifestyle concerns.

Several platforms have been established by the MOH in Saudi Arabia following the COVID-19 pandemic to enhance access to healthcare services. In this study, some HF participants reported difficulties adhering to HF self-care management during the COVID-19 pandemic. In contrast to a previous study by Tash and Al-Bawardy, the collaboration between the Saudi Health Council, ABSHER, the national cardiovascular registry established in 2015, and platforms such as Tawakkalna and Sehhaty, launched during the SARS-CoV-2 pandemic, is widely used for vaccination and health information in Saudi Arabia [22]. These platforms can support compliance among all HF patients undergoing treatment and enable them to consult a doctor through virtual appointments; however, clinicians should closely assess each patient’s complaints. Some participants in this study found that virtual appointments did not offer any benefits during the COVID-19 pandemic, despite frequent delays in appointments and a lack of medication being supplied. This finding aligns with a study by Bezerra et al., which reported that a lack of digital literacy and lower interest in using new technology, especially for HF self-management, are prevalent among HF patients [23]. Virtual consultation with the physician encompasses an examination of current prescription medications and laboratory findings. Contrary findings were reported by another study by Tash and Al-Bawardy, which stated that a “Seha” virtual hospital has been operational since 2020, an initiative fast-tracked by the COVID-19 pandemic, as Saudi leadership has adopted an aggressive approach, featuring a central cardiac imaging and cardiovascular information center to discuss data and complex cases with the National Virtual Heart committee [22]. This initiative, launched by Saudi Arabia’s MOH, has improved access to healthcare services and increased patient satisfaction with treatment.

Fewer cardiac rehabilitation programs are being developed in Saudi Arabia, in line with Saudi Vision 2030, which promotes lifestyle education, psychosocial support, self-care management, and exercise training. These initiatives benefit patients with heart disease and support overall health improvement but still need greater public awareness. This aligns with other studies. Skouri et al. reported that the Middle East faces several challenges, including a lack of awareness among physicians, the absence of structured HFPs, a shortage of national strategies for cardiac rehabilitation, and a limited number of specialized clinics run by certified cardiologists [21]. Saudi Arabia has numerous initiatives and campaigns, as stated by Tash and Al-Bawardy, aimed at promoting strategies and policies that encourage a heart-healthy lifestyle and a nutritious diet; however, these have yet to be widely adopted and enforced, such as the city humanization and walkability campaign [22]. Offering these support groups and programs to HF patients allows them to exchange views and experiences and encourages participation in social activities.

Limitations

This study has several limitations and challenges. The first relates to the recruitment of participants. The current study was conducted in the present setting, primarily among Saudi participants, as including a diverse group of participants diagnosed with HF could have compromised the validity of the research findings. A significant number of expatriates live in the Kingdom of Saudi Arabia, and they may have different perceptions of HF due to their immigrant status. Future studies on cultural diversity in HF self-care management are recommended to obtain more valid results.

Second, there are fewer studies conducted in Saudi Arabia regarding HF. This study did not include the perspectives of individuals who are directly involved in the social network of HF patients, such as caregivers and members of the HCP team. These individuals can also serve as valuable sources of support for patients with HF.

Third, another limitation includes the minimal participation of females in this study. Most of the participants were men. The primary reason for the limited number of female participants was that most patients admitted to the selected units, specifically the Coronary Care Unit and the Cardiac Ward, were male. Since this study setting only covered these two units, it is possible to extend the selected units to another unit, the Cardiac Thoracic Surgical ICU, and other units as well. Future research should consider collecting data from participants at the Cardiac Centre.

Furthermore, it is important to consider cultural sensitivities and ethical considerations when reflecting on the challenges faced as an expatriate researcher in this study. These include navigating gender segregation and being sensitive to local customs. Since strict gender roles are observed, careful planning is needed to ensure smooth and respectful cultural adaptation while conducting research in Saudi Arabia. Language barriers also exist due to varying styles of polite communication cues and body language; consequently, the researcher may encounter certain difficulties during interviews.

## Conclusions

In summary, the findings of this study emphasize the importance of support systems as a foundation for self-care management among patients with HF. Family caregivers, HCPs, and institutional resources all play critical roles in ensuring comprehensive self-care for patients with HF. Participants with HF reported that their spouses were their primary caregivers. Most were satisfied with the care and communication provided by HCPs. Many did not exercise because of their condition. The government covered most reported HF-related costs, and some individuals received financial aid. Others relied on family and relatives for support. The findings of this study have important implications for HCP teams. Cardiologists, followed by physicians, nurses, respiratory therapists, and other HCPs, should play a vital role in assessing patients’ knowledge, readiness for self-care or acceptance of their condition, and mental health status. Moreover, HCPs need to intervene early to provide optimal care for patients with HF. The current healthcare system mainly focuses on clinical outcomes, paying less attention to individual needs and preferences. Therefore, discharge education should be revised to involve family members or caregivers and provide structured counseling on medication review, diet, exercise, and warning signs or red flags while addressing patients’ health literacy. This approach can help ensure that each patient’s individual needs and emotional well-being are prioritized in self-care management after discharge.

The findings of this study aim to address poor self-care management outcomes observed among patients with HF in Saudi Arabia, where comprehensive health promotion and lifestyle behavioral modification among Middle Eastern populations may be compromised. This study may help patients understand how they adapt to changing conditions and develop emotional coping strategies when managing the distressing symptoms of HF in their daily lives. Moreover, these findings may benefit patients with HF by helping them overcome the challenges associated with HF, enhance their self-care management, and improve adherence to treatment. Social media, newspapers, educational initiatives, and free health screenings may play a significant role in raising public awareness and understanding of risk factors for HF.

## Appendices

Appendix A

**Table 2:** Data collection form.

Data collection form Date: ________________ Initials of person completing form: _______ Hospital: KFMMC, Dharan. Location: Coronary care unit/Cardiac ward	نموذج جمع بيانات التاريخ: __________________ الأحرف الأولى من اسم الشخص الذي يكمل النموذج: ___________________ المستشفى: مجمع الملك فهد الطبي العسكري بالظهران المكان: وحدة العناية التاجية / قسم القلب
Research title: Understanding of heart failure self-care management from the perspective of heart failure patients	عنوان البحث: فهم إدارة الرعاية الذاتية لقصور القلب من منظور مريض قصور القلب
1. Patient-identifying information. Age: _____ Sex: ___ Height: ____ Weight: _____ Employment status: _______ Nationality: ______ Marital Status: _____ Educational level: _________ Immunization status: __________	١. معلومات هوية المريض العمر: _____ الجنس: _____ الطول: _ الوزن: ________ الوضع الوظيفي: _________الجنسية:_____ الحالة الاجتماعية:___المستوى: التعليمي:____حالة التطعيم: _______
2. Communication and socioeconomic assessment. Language spoken: _______ Interpreter required: ____________ Living arrangement: _______ Economic status: ______ Watcher: History of readmission: ________ Contact person: Name/Relationship/H/p number: ________	٢. تقييم التواصل و التقييم الاقتصادي الاجتماعي: اللغة التي يتحدث بها:_____________ يتطلب مترجم:_______ __ ترتيب المعيشة:______ الوضع الاقتصادي:____ __مراقب:___________ تاريخ دخول المستشفى مرة أخرى:_______________ الشخص الذي يمكن الاتصال به: 1) الإسم / صلة القرابة / رقم التواصل: 2) الإسم / صلة القرابة / رقم التواصل:
3. Health history. Past medical history: _______ Diagnosis: _______ Infectious disease: Blood transfusion history:	٣. التاريخ الصحي / السجل الصحي: التاريخ الطبي الماضي: التشخيص: الأمراض المعدية: سجل نقل الدم:
4. Drug history. Any drug prescriptions before: _______	٤. سجل الأدوية: أي وصفات دوائية سابقة:
5. Medications. List of current medications: _______	٥. الأدوية: قائمة الادوية الحالية:
6. Laboratory investigations information. Any abnormal results: _______	٦. معلومات الفحوص المختبرية: هل هناك وجود لنتائج غير طبيعية:

Appendix B

**Table 3:** Semi-structured interview guide based on the Adams study

Duration	Objective/item	Questions	Probe	Statement
Five minutes	Introduction	Assalamualaikum wbt. My name is Noor Ezah, and I am a master's nursing student at IIUM. I would like to interview you for my research study.	Introduce self to participants	Rapport
15 minutes	Objective 1: To explore the perception of heart failure patients toward the disease, treatment, and its related effects	(1) General introduction: Could you tell me about your health problems, heart failure? Can you describe your perception of heart failure as a patient, toward the disease, treatment, and its related effects? (2) Burden of treatment: How do you experience self-management of your disease, thinking about this burden of treatment? (3) Illness perception: How much does your illness affect your life, physically, mentally, and emotionally?	Experience	-
15 minutes	Objective 2: To explore how heart failure patients perceive heart failure self-care management	Can you explain the barriers and facilitators toward heart failure self-care management? (1) Daily management of the disease: What do you have to do to take care of yourself and the disease? How do you cope in daily life with self-management of the disease? Do you sometimes feel that it is hard to do all these things to take care of your health? Do you ever skip any of the things you should do? (2) Capacity: How do you perceive your capacity in daily life? What are you doing on a daily basis to manage your condition? (3) Readmission (if related): How do you think you became sick enough to be readmitted to the hospital? Before you left the hospital last time, did you have a scheduled appointment with your doctor? When you or your family members encountered problems/concerns after you left the hospital, did you know who to call? Were the discharge instructions written down easy to read and understand?	Stigma/challenges	-
20 minutes	Objective 3: To explore the needs of self-care management among heart failure patients and how the needs are being supported by caregivers and HCPs	Interpersonal relations needs and support: (1) Caregiver support: Tell me about the person who’s taking care of you, your diet, and medicines that you take. Think about the last time that you had symptoms. Can you describe what you did? What does the caregiver do to prevent worsening HF symptoms? (2) Healthcare professionals support: What information and instructions have you received? (3) Emotional support: Is this something you can relate to? Is there something you do to keep your spirit up? (3) Social support: In relation to your HF, do you get any help from others?	Solution/support	-
Five minutes	Closing	Thank you for spending your time and being willing to share your understanding of heart failure self-care management from the perspective of heart failure patients and be a part of my participants for this research project. Is there something important you want to add?	Thank the participant	-
Total: 60 minutes (one hour)			-	-
